# Radiomics analysis based on CT for the prediction of pulmonary metastases in ewing sarcoma

**DOI:** 10.1186/s12880-023-01077-4

**Published:** 2023-10-02

**Authors:** Ying Liu, Ping Yin, Jingjing Cui, Chao Sun, Lei Chen, Nan Hong, Zhentao Li

**Affiliations:** 1 Department of Radiology, Peking University People’s Hospital, 11 Xizhimen Nandajie, Xicheng District, Beijing, 100044 People’s Republic of China; 2 United Imaging Intelligence (Beijing) Co., Ltd, Yongteng North Road, Haidian District, Beijing, 100094 People’s Republic of China

**Keywords:** Ewing sarcoma, Radiomics, Pulmonary metastases, Computed tomography

## Abstract

**Objectives:**

This study aimed to develop and validate radiomics models on the basis of computed tomography (CT) and clinical features for the prediction of pulmonary metastases (MT) in patients with Ewing sarcoma (ES) within 2 years after diagnosis.

**Materials and methods:**

A total of 143 patients with a histopathological diagnosis of ES were enrolled in this study (114 in the training cohort and 29 in the validation cohort). The regions of interest (ROIs) were handcrafted along the boundary of each tumor on the CT and CT-enhanced (CTE) images, and radiomic features were extracted. Six different models were built, including three radiomics models (CT, CTE and ComB models) and three clinical-radiomics models (CT_clinical, CTE_clinical and ComB_clinical models). The area under the receiver operating characteristic curve (AUC), and accuracy were calculated to evaluate the different models, and DeLong test was used to compare the AUCs of the models.

**Results:**

Among the clinical risk factors, the therapeutic method had significant differences between the MT and non-MT groups (*P*＜0.01). The six models performed well in predicting pulmonary metastases in patients with ES, and the ComB model (AUC: 0.866/0.852 in training/validation cohort) achieved the highest AUC among the six models. However, no statistically significant difference was observed between the AUC of the models.

**Conclusions:**

In patients with ES, clinical-radiomics model created using radiomics signature and clinical features provided favorable ability and accuracy for pulmonary metastases prediction.

## Introduction

Ewing sarcoma (ES) is a highly aggressive bone sarcoma, that occurs in any age with a peak incidence in children and young adults. The most powerful adverse prognostic factor across different treatment strategies in ES is metastases, with the lungs and bone being the most common metastatic locations. At presentation, approximately 20–25% of patients show metastases, usually affecting the lungs (70–80%) and the bone (40–45%) [1, 2]. At present, the overall survival (OS) of patients with localized disease has improved remarkable by multimodal treatment, with a 10-year rate of 55–65%. However, the suboptimal outcome in patients with pulmonary metastases with 2- to 10-year event-free survival (EFS) of 30–36% still remains to be a great concern [1, 3].

Computed tomography (CT) is widely used for diagnosing pulmonary metastases as a non-invasive and reliable method. However, because micro-metastases are not identified by the current radiological techniques, improving the accuracy in detecting pulmonary metastases at the time of diagnosis is necessary [4]. Radiomics analysis, which refers to extracting radiomics features from medical images and then transferring them into high-dimensional data, has been applied in various types of tumors for diagnosis and prognosis and as a treatment response imaging biomarker [5–7]. Few studies have employed radiomics to identify patients at risk for developing pulmonary metastases [8, 9], and Dai et al. used multiparametric magnetic resonance imaging (MRI)-based radiomic analysis for the distinction of ewing sarcoma and osteosarcoma [10]. However, to the best of the author’s knowledge, no studies have been reported on the use of radiomics to predict pulmonary metastases in ES.

This study aimed to develop and validate radiomics models based on CT to predict pulmonary metastases in patients with ES, which could be a potential tool for guiding more personalized medicine.

## Materials and methods

### Patients

**Fig. 1:**
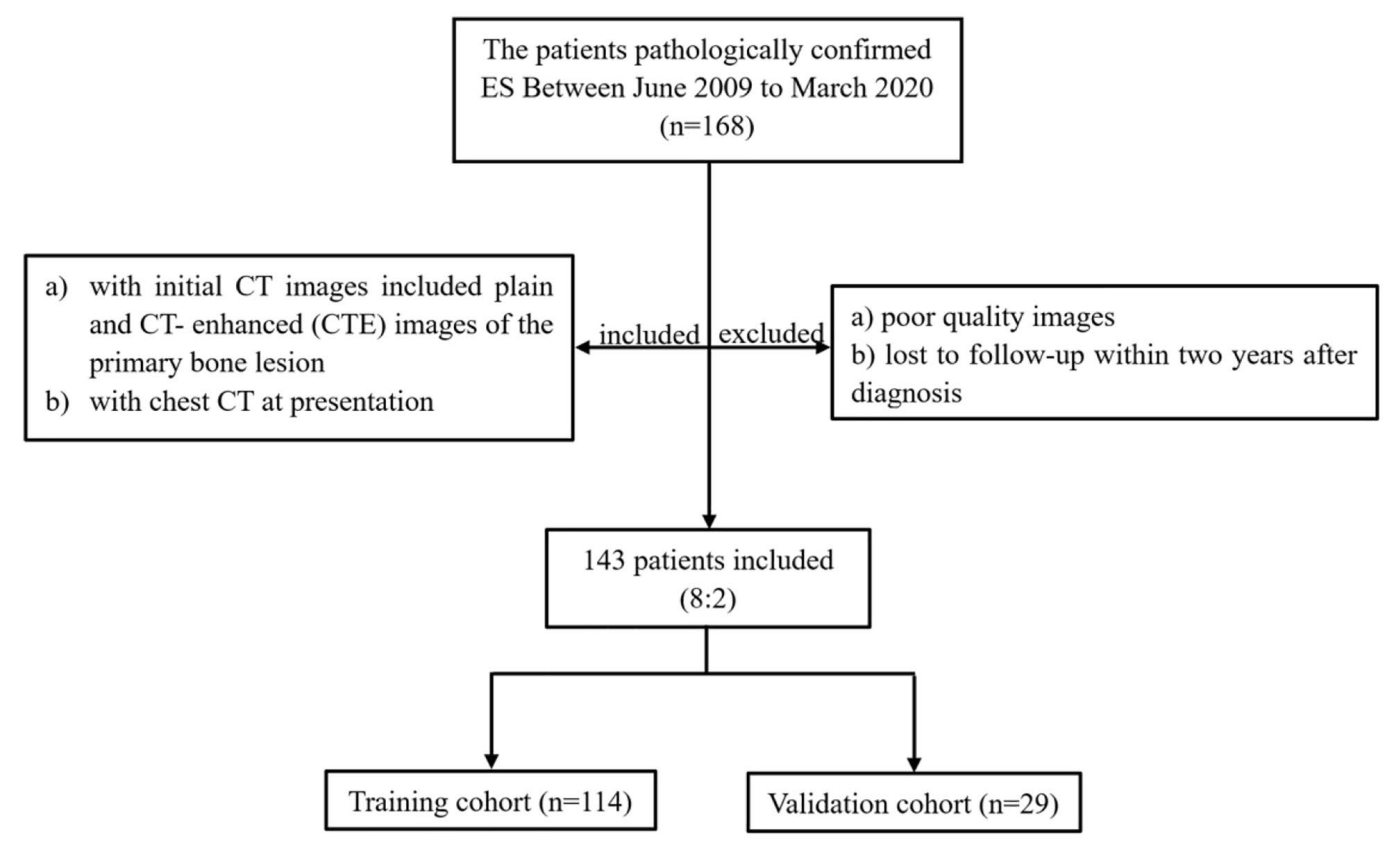
Flowchart of the patients selection process in our study

This retrospective study was approved by the Ethics Committee of our hospital, and the need for a written informed consent from patients was waived. The inclusion criteria were as follows: (a) histopathologically-confirmed ES by biopsy or tumor resection; (b) with initial CT images, including plain and CT- enhanced (CTE) images of the primary bone lesion, and chest CT at presentation. The exclusion criteria were as follows: (a) poor quality images, which were inadequate for the following analysis; (b) without follow-up of 2 years after diagnosis. The patients were randomly divided into training cohort and validation cohort at a ratio of 8:2[11], the training cohort was used for feature selection and models building, and the validation cohort was used for evaluation. Figure 1 shows the flow diagram of the recruitment process.

All patients underwent follow-up of more than 2 years after diagnosis, and enhanced chest CT were monitored once every 3 months. The patients who suffered pulmonary metastases of ES within 2 years after diagnosis were defined as the presence (MT) cohort, and those without any suspicious nodules on chest CT images during the 2-year follow-up after diagnosis were defined as the absence (non-MT) cohort. The MT group were those patients with multiple nodules at presentation or nonspecific nodules that increased in number or size, as detected by chest CT during the 2-year follow-up. The potential clinical risk factors that may be associated with pulmonary metastases were obtained from medical records as follows: gender, age, tumor location (pelvic bone, long bone or other locations), lactate dehydrogenase (LDH) level, alkaline phosphatase (ALP) level, major length and therapeutic method.

### Imaging acquisition and analysis

The CT and CTE images were obtained from the picture archiving and communication system (PACS). CT images were acquired using multidetector row CT (MDCT) systems (Brilliance iCT, Philips Healthcare, Best, the Netherlands; Light Speed Volume CT, GE Healthcare, Waukesha, USA), with the following parameters: 120 kV, 240 to 260 mAs, collimations of 64 × 0.6 mm, and slice thicknesses of 5 mm. CTE acquisition was performed after a 70s delay following intravenous administration, with 1.5 mL/kg iodinated contrast (100 mL of 370 mg J/mL iopromide; Bayer Schering Pharma, Berlin, Germany) by using an automatic pump injector (Ulrich CT Plus 150, Ulrich Medical) at a rate of 2.5 mL/s through the antecubital vein.

The regions of interest (ROIs) were handcrafted along the boundary of each tumor on each slice by using uAI Research Portal [12] (Shanghai United Imaging Intelligence Co., Ltd, Shanghai, China) by two experienced radiologists (YL and PY, with 6 and 10 years of CT experience, respectively). The ROIs were delineated on both the CT and CTE images by consensus of the two radiologists.

### Radiomics feature extraction and selection

Z-score normalization method was implemented in CT images to minimize the intensity discretization before feature extraction. A total of 2600 radiomics features were extracted using Python software (https://www.python.org), based on algorithms provided in Pyradiomics [13] (version 2.1.1). The features could be further divided into three groups: intensity, tumor shape and texture features. Maximum normalization of absolute scaler method was applied to normalize the features before feature selection and model building. The univariate method - Analysis of Variance (ANOVA) and the multivariate method-least absolute shrinkage and selection operator (LASSO) algorithms were applied to select the most valuable radiomics features [14].

## Model construction

After feature selection was conducted, radiomics and clinical-radiomics models were built to predict the pulmonary metastases of ES. The radiomics model incorporated only radiomics features, and then the clinical risk factors (therapeutic method, *P*＜0.05) were introduced to build the clinical-radiomics model by the stepwise multiple logistic regression. Gaussian process (GP), logistic regression (LR) and partial least squares discrimination analysis (PLS-DA) classifiers with high stability were investigated.

### Model assessment

The training cohort was used for feature selection and model construction, and the validation cohort was used for model evaluation. The predictive performance of the different models was assessed using the receiver operating characteristics (ROC) curve. The area under the curve (AUC), accuracy (ACC), sensitivity, and specificity were also reported for the radiomics and clinical-radiomics models. The comparisons between AUCs of the models were conducted by using DeLong test.

### Statistical analysis

All statistics were performed using SPSS 22.0 (IBM Corp, NY, USA), with *P* < 0.05 considered as statistically significant. Comparisons of patient continuous clinical risk factors were conducted by t-test, and χ2 test or Fisher’s exact test was applied for categorical variables.

## Results

### Clinical characteristics

Finally, a total of 143 patients with (MT, n = 76) and without (non-MT, n = 67) pulmonary metastases were recruited in this study. The clinical characteristics are shown in Table 1.

Among all the cases, 85 lesions were located in pelvic bone, 39 lesions were located in the bones of extremities, and 19 were located in other areas (7 in clavicle, 5 in scapula, 3 in spine, 3 in rib, and 1 in calcaneus). No significant difference were found in gender, age, tumor location, LDH level, ALP level and major length between the MT and non-MT groups (*P*＞0.05), except for the therapeutic method (*P*＜0.05).

### Predictive performance of radiomics model

By using ANOVA and LASSO methods, 10, 27 and 15 optimal features were ultimately selected as feature groups for individual CT, CTE and the combination of CT and CTE (ComB) models, respectively.

**Fig. 2:**
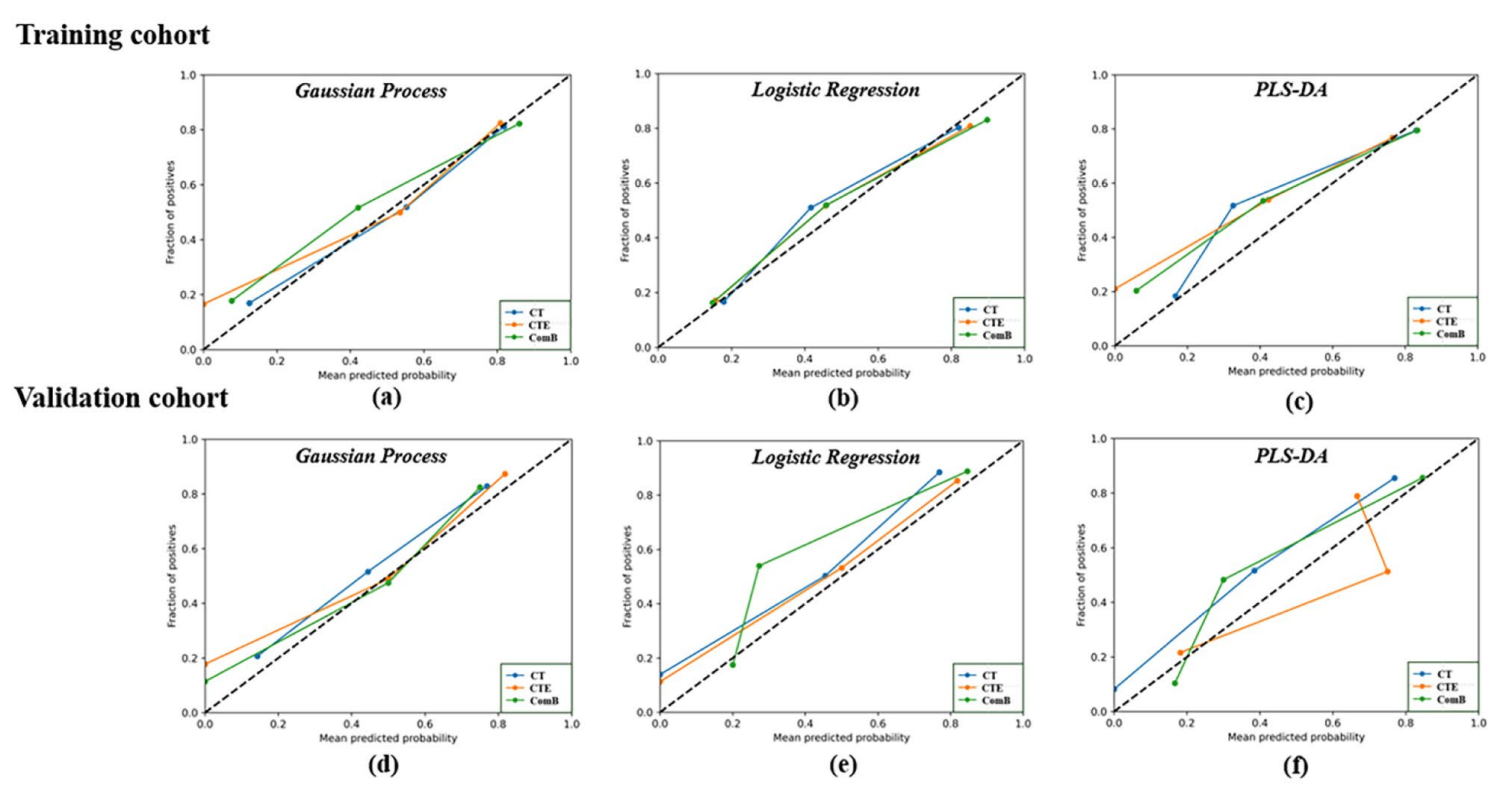
Calibration curves of the three radiomics models in the training and validation cohort. **(a)**: Calibration curves of the gaussian process based radiomics models (CT model, CTE model and ComB model) in the training cohort; **(b)**: Calibration curves of the logistic regression based radiomics models (CT model, CTE model and ComB model) in the training cohort; **(c)**: Calibration curves of the PLS-DA based radiomics models (CT model, CTE model and ComB model) in the training cohort; **(d)**: Calibration curves of the gaussian process based radiomics models (CT model, CTE model and ComB model) in the validation cohort; **(e)**: Calibration curves of the logistic regression based radiomics models (CT model, CTE model and ComB model) in the validation cohort; **(f)**: Calibration curves of the PLS-DA based radiomics models (CT model, CTE model and ComB model) in the validation cohort

**Fig. 3:**
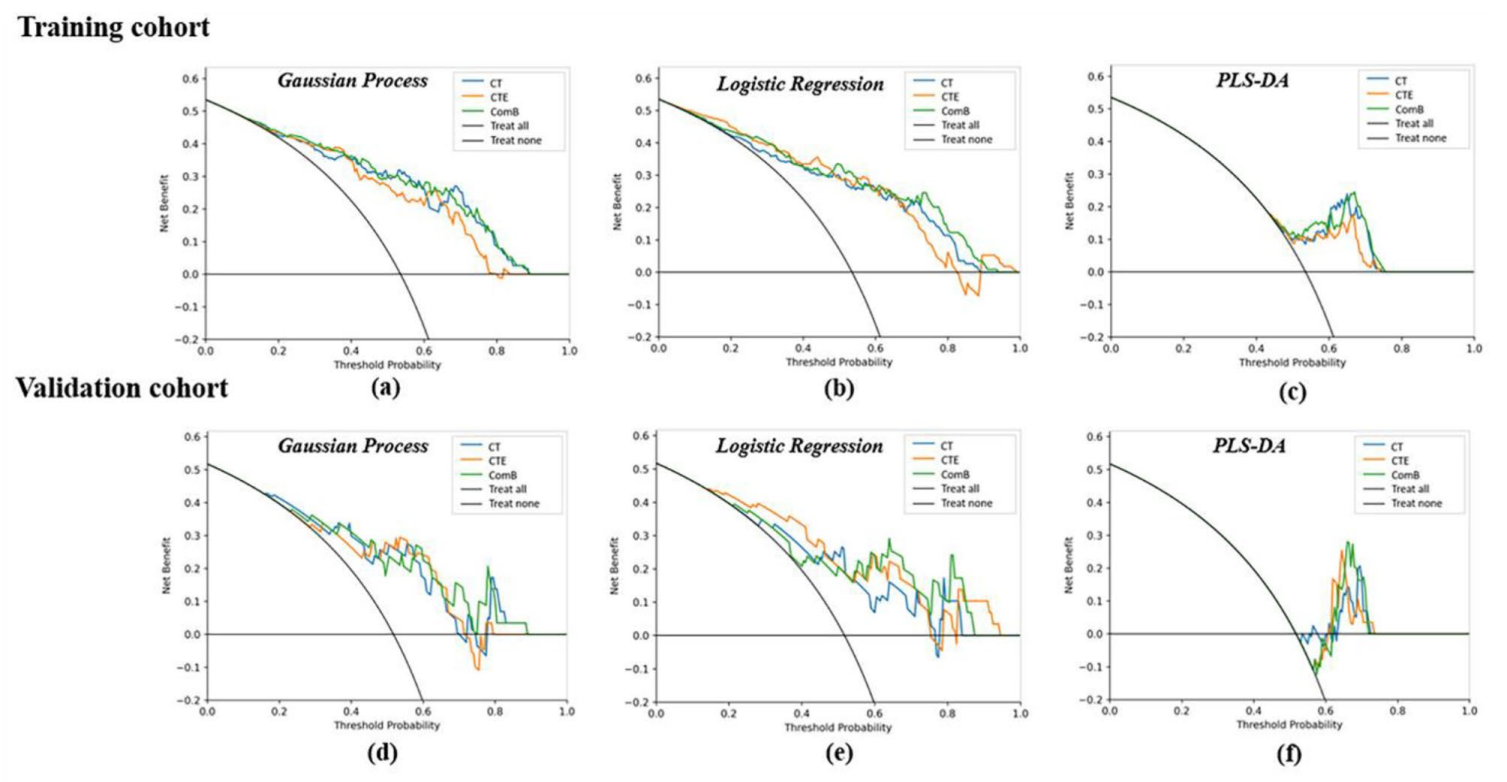
The decision curve analysis (DCA) of the three radiomics models in the training and validation cohort. **(a)**: The DCA of the gaussian process based radiomics models in the training cohort. **(b)**: The DCA of the logistic regression based radiomics models in the training cohort. **(c)**: The DCA of the PLS-DA based radiomics models in the training cohort. **(d)**: The DCA of the gaussian process based radiomics models in the validation cohort. **(e)**: The DCA of the logistic regression based radiomics models in the validation cohort. **(f)**: The DCA of the PLS-DA based radiomics models in the validation cohort. The y-axis measures the net benefit

The ComB model had the highest AUC of 0.829, 0.852 and 0.848 among the models in the validation cohort by using GP, LR and PLS-DA classifiers respectively. The AUC, ACC, sensitivity and specificity of the CT, CTE, and ComB models are presented in Table 2. Figure 2 illustrates the calibration of the radiomics models, and the results showed good calibration in the training and validation cohorts. The decision curve analysis (DCA) for the three radiomics models of different classifiers is exhibited on Fig. 3, which showed the three radiomics models had similar benefit in the threshold probability.

**Table 1:** The clinical characteristics of patients

	MT	Non-MT	χ^2^/t value	*P* value
Gender			χ^2^ = 0.478	0.489
Male	48	46		
Female	28	21		
Age(year)			t = 0.011	0.919
Mean ± SD	19.49 ± 10.34	16.79 ± 11.16		
Tumor Location			χ^2^ = 3.164	0.206
Pelvic bone	49	36		
Long bone	16	23		
Other locations	11	8		
LDH level			χ^2^ = 1.162	0.281
Normal	48	48		
Abnormal	28	19		
ALP level			χ^2^ = 0.660	0.417
Normal	70	59		
Abnormal	6	8		
Major length			t = 0.831	0.364
Mean ± SD	92.93 ± 32.19	85.11 ± 30.89		
Therapy Method			χ^2^ = 8.434	**0.004**
Surgery and chemotherapy	53	60		
Chemotherapy	23	7		

The bold font means *P*＜0.05

**Table 2:** The value of Different Radiomics Models in Training cohort and Validation cohort

				Train						Validation	
	AUC	ACC	95%CI	Sensitivity	Specificity		AUC	ACC	95%CI	Sensitivity	Specificity
Pre-CT											
GP	0.865	0.798	0.805–0.914	0.803	0.792		0.805	0.793	0.635–0.924	0.867	0.714
LR	0.842	0.781	0.779–0.895	0.754	0.811		0.800	0.689	0.635–0.913	0.667	0.714
PLS-DA	0.834	0.771	0.769–0.891	0.721	0.830		0.819	0.724	0.656–0.928	0.733	0.714
CTE											
GP	0.831	0.754	0.765–0.890	0.754	0.736		0.824	0.689	0.657–0.948	0.733	0.786
LR	0.834	0.789	0.761–0.891	0.787	0.792		0.833	0.759	0.678–0.944	0.800	0.714
PLS-DA	0.796	0.737	0.723–0.856	0.738	0.736		0.800	0.586	0.637–0.919	0.733	0.857
ComB											
GP	0.868	0.781	0.808–0.917	0.705	0.868		0.829	0.689	0.678–0.933	0.800	0.786
LR	0.866	0.798	0.807–0.913	0.852	0.736		0.852	0.724	0.709–0.956	0.800	0.857
PLS-DA	0.845	0.754	0.781–0.898	0.721	0.755		0.848	0.793	0.697–0.957	0.800	0.857

Note. AUC area under the receiver-operating characteristic curve, ACC accuracy, GP gaussian process, LR logistic regression, PLS-DA partial least-squares discrimination analysis, CT computed tomography, CTE CT-enhanced, ComB, the combination of CT and CTE features

### Predictive performance of clinical-radiomics model

**Fig. 4:**
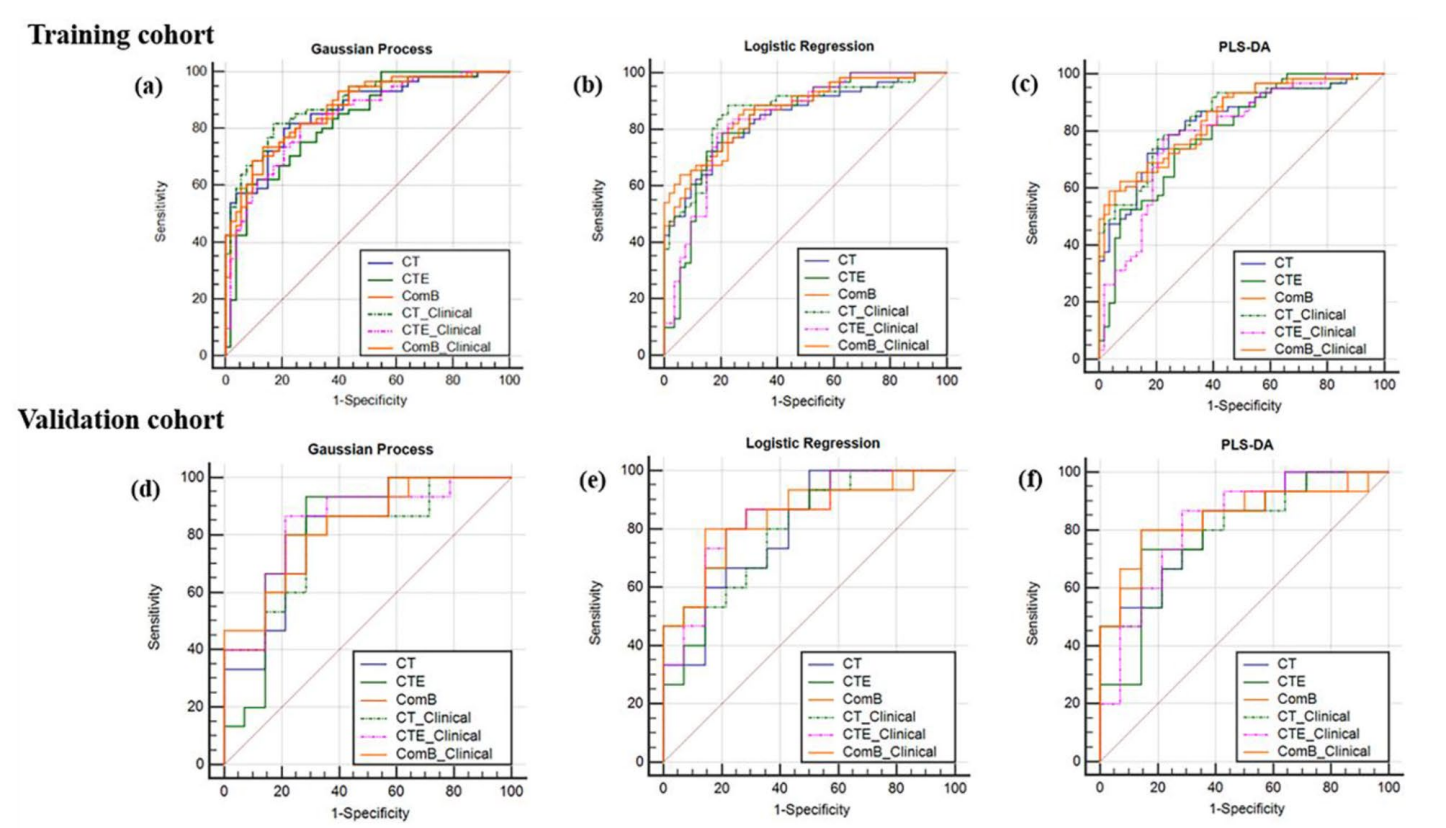
The ROC curves of the radiomics and clinical-radiomics models. **(a)-(c)**, the ROC curves of radiomics and clinical-radiomics models in training cohort, by using GP, LR and PLS-DA classifiers, respectively; **(d)-(f)**, the ROC curves of radiomics and clinical-radiomics models in validation cohort, by using GP, LR and PLS-DA classifiers, respectively

For GP and LR classifiers, the CTE_clinical model had the highest AUCs of 0.843 and 0.843 in the validation cohort, respectively. Whereas the ComB_clinical model achieved the highest AUC of 0.843 in the validation cohort by using the PLS-DA classifier. Table 3 shows the AUC, ACC, sensitivity and specificity of the three clinical-radiomics models. The ROC curves of the radiomics and clinical-radiomics models are exhibited in Fig. 4. There were no statistical differences between the AUC values of the models.

**Table 3:** The value of Different Clinical-Radiomics Models in Training cohort and Validation cohort

	Train							Validation				
	AUC	ACC	95%CI	Sensitivity	Specificity			AUC	ACC	95%CI	Sensitivity	Specificity
CT _ clinical												
GP	0.887	0.824	0.833–0.933	0.820	0.831			0.795	0.793	0.635–0.923	0.867	0.714
LR	0.862	0.833	0.798–0.914	0.885	0.774			0.810	0.724	0.639–0.916	0.800	0.643
PLS-DA	0.848	0.781	0.782–0.903	0.770	0.792			0.800	0.724	0.636–0.914	0.733	0.714
CTE _ clinical												
GP	0.840	0.781	0.773–0.896	0.820	0.736			0.843	0.828	0.697–0.952	0.867	0.786
LR	0.835	0.798	0.760–0.894	0.787	0.811			0.843	0.793	0.692–0.949	0.733	0.857
PLS-DA	0.795	0.772	0.717–0.860	0.770	0.773			0.824	0.793	0.657–0.938	0.867	0.714
ComB _ clinical												
GP	0.872	0.798	0.812–0.919	0.738	0.868			0.824	0.759	0.676–0.929	0.800	0.714
LR	0.869	0.781	0.811–0.919	0.803	0.736			0.833	0.793	0.688–0.938	0.800	0.786
PLS-DA	0.852	0.763	0.789–0.905	0.738	0.717			0.843	0.828	0.697–0.952	0.800	0.857

Note. AUC area under the receiver-operating characteristic curve, ACC accuracy, GP gaussian process, LR logistic regression, PLS-DA partial least-squares discrimination analysis, CT computed tomography, CTE CT-enhanced, ComB, the combination of CT_clinical and CTE_clinical models

**Fig. 5:**
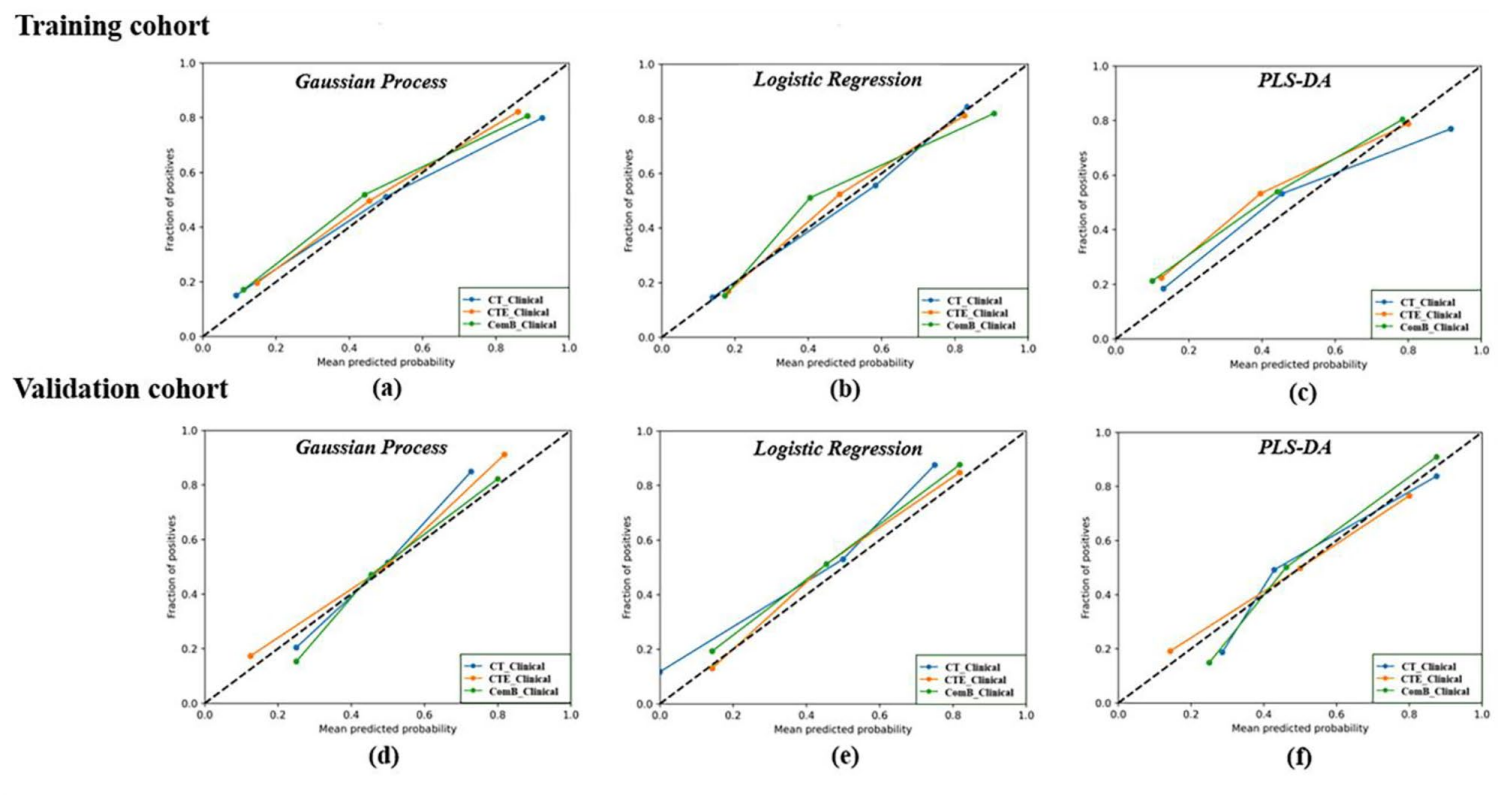
Calibration curves of the three clinical-radiomics models in the training and validation cohort. **(a)**: Calibration curves of the gaussian process based clinical-radiomics models (CT_clinical model, CTE_clinical model and ComB_clinical model) in the training cohort;**(b)**: Calibration curves of the logistic regression based clinical-radiomics models (CT_clinical model, CTE_clinical model and ComB_clinical model) in the training cohort; **(c)**: Calibration curves of the PLS-DA based clinical-radiomics models (CT_clinical model, CTE_clinical model and ComB_clinical model) in the training cohort; **(d)**: Calibration curves of the gaussian process based clinical-radiomics models (CT_clinical model, CTE_clinical model and ComB_clinical model) in the validation cohort; **(e)**: Calibration curves of the logistic regression based clinical-radiomics models (CT_clinical model, CTE_clinical model and ComB_clinical model) in the validation cohort; **(f)**: Calibration curves of the PLS-DA based clinical-radiomics models (CT_clinical model, CTE_clinical model and ComB_clinical model) in the validation cohort

**Fig. 6:**
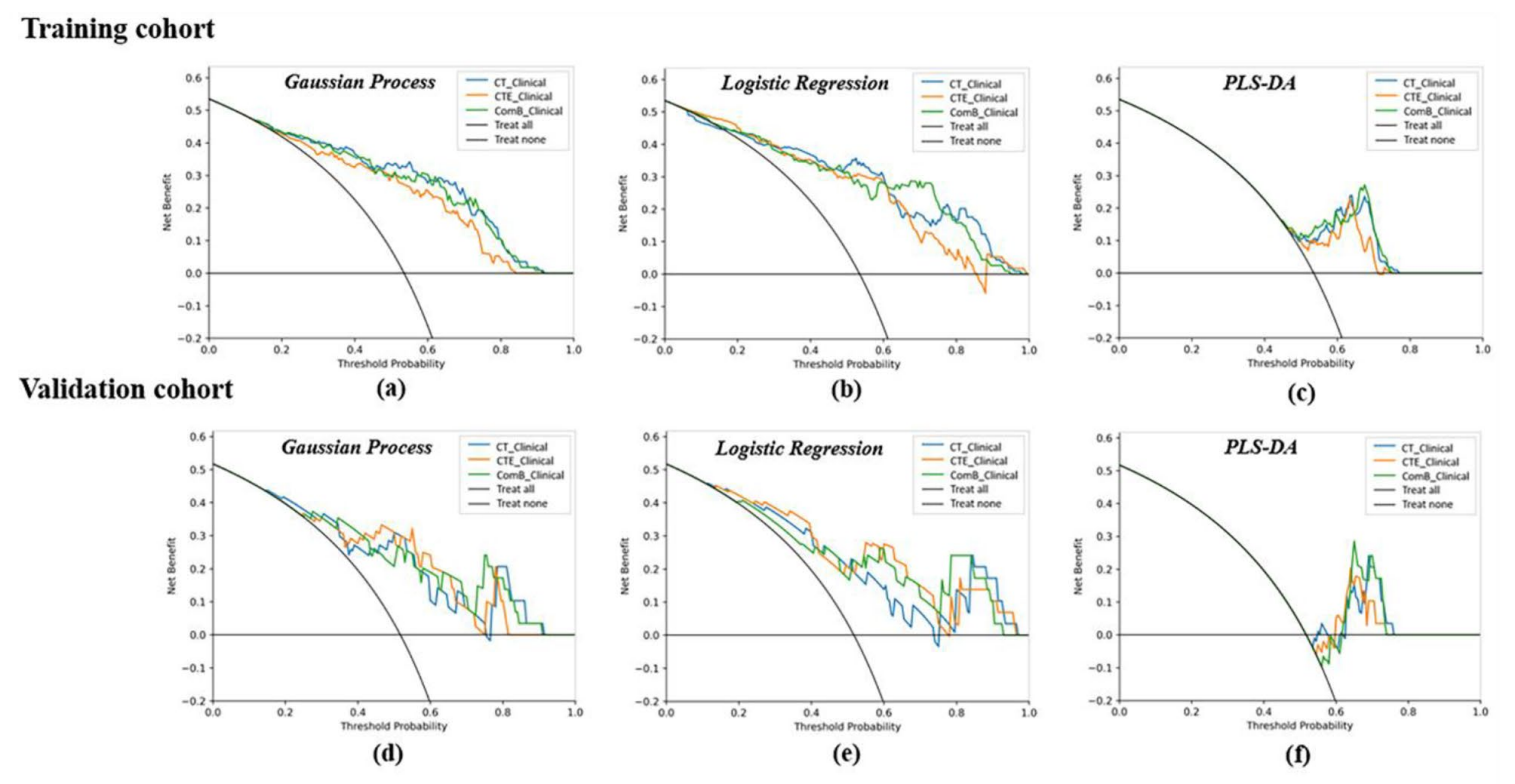
The decision curve analysis (DCA) of the three clinical-radiomics models in the training and validation cohort. **(a)**: The DCA of the gaussian process based clinical-radiomics models in the training cohort. **(b)**: The DCA of the logistic regression based clinical-radiomics models in the training cohort. **(c)**: The DCA of the PLS-DA based clinical-radiomics models in the training cohort. **(d)**: The DCA of the gaussian process based clinical-radiomics models in the validation cohort. **(e)**: The DCA of the logistic regression based clinical-radiomics models in the validation cohort. **(f)**: The DCA of the PLS-DA based clinical-radiomics models in the validation cohort. The y-axis measures the net benefit

The calibration curve of the clinical-radiomics models showed good calibration results in the training and validation cohorts (Fig. 5). The DCAs of the three clinical-radiomics models indicated that these models had similar benefit in the threshold probability by using the GP, LR and PLS-DA classifiers (Fig. 6).

## Discussion

To the best of the author’s knowledge, this study was the first to predict the risk of developing pulmonary metastases in patients with ES on the basis of CT radiomics features, in a 2-year follow-up period after diagnosis. The clinical-radiomics models based on combined features and the radiomics models demonstrated similar performance, and the sensitivity and specificity of the clinical-radiomics models and radiomics models were excellent.

Among the clinical risk factors, gender, age, tumor location, LDH level, ALP level and the major length of the tumor were confirmed to be not the prognostic factors for the occurrence of pulmonary metastases, without statistical difference between the MT and non-MT groups. By contrast, therapeutic method showed correlation with pulmonary metastases. Some previous studies reported that gender was not a risk factor for pulmonary metastases of ES, larger tumors had a higher chance of pulmonary metastases, and the results of age, and primary location were controversial. Li et al. found that surgery was a protective factor against pulmonary metastases and chemotherapy had no significant difference between MT and non-MT groups [15–18].

In the past decades, radiomics as a promising methodology has been widely used in the diagnosis, differentiation, staging and monitoring of tumors, owing to the progress in extracting vital high throughout analysis features and screening large numbers of features. In prior studies, radiomic analysis has been identified as a powerful method for the predicting pulmonary metastases of soft tissue and bone sarcomas [19–21].

For prediction of pulmonary metastases in ES, radiomics features based on CT were used to develop and validate three different radiomics models, which achieved high performance. Among the three models, the ComB model presented the highest performance in the validation set by using the LR classifier, but there were no statistical difference was observed between the AUCs of the models. Three well-known classifiers were used in this study, including GP, LR and PLS-DA. As the most widely used method in the past studies related to radiomics analysis, LR is a stratification algorithm suitable for analyzing large data sets of features in small samples, and designed to avoid overfitting and predict the class probability of a given categorical dependent variable [22]. GP is a nonparametric method that is based on Laplace approximation used for classification and regression, and it could handle various problems such as the curse of dimension, complex data types and insufficient capacity of the classical linear method [23, 24]. And PLS-DA method has been applied in previous studies, is demonstrated to be able to deal with high-dimensional radiomics dataset [25].

Furthermore, three clinical-radiomics models were established for patients with ES. The CTE_clinical and ComB_clinical models offered preferable prognostic ability (AUC = 0.843/0.843, in validation cohort) in the prediction of pulmonary metastases, and there were no statistic differences between the AUCs of the clinical-radiomics models. The prediction ability of the three clinical-radiomics models were not markedly enhanced relative to that of the radiomics models, and such results may be partially due to the sample size being relatively small and only one clinical risk factor being combined in the clinical-radiomics models.

There are several limitations in the retrospective study. First, due to the low incidence rate of ES, the patient population was relatively small, with all patients coming from one single center, and internal validity was used rather than external validity. Therefore, large-scale studies with external validation are required before widespread implementation of the models in the clinical practice. Second, most cases were diagnosed by surgical specimens, but a small fraction of the pathological diagnosis was obtained by biopsy, which could result in some bias. Third, multiparametric MR images with better soft-tissue resolution should be implemented in future studies to improve the precision and robustness of the models. Forth, manual segmentation is time consuming, and it can’t deliver reproducible results. But manual segmentation was used as a gold standard, and it has been applied in many previous studies and yielded excellent results. Fifth, the relationship between the radiomic analysis of the primary tumor and the possibility to develop pulmonary metastases was unclear, and this need more research in future.

In summary, the CT based radiomics model was effective in predicting the pulmonary metastases in patients with ES, and thus could be a potential tool for the accurate risk stratification and precision medicine.

## Data Availability

The datasets generated during and/or analysed during the current study are available from the corresponding author on reasonable request.

## Abbreviations

ES: Ewing sarcoma
OS: overall survival
EFS: event-free survival
CT: Computed tomography
CTE: CT- enhanced
LDH: lactate dehydrogenase
ALP: alkaline phosphatase
PACS: picture archiving and communication system
ROIs: Regions of interest
ANOVA: Analysis of variance
LASSO: Least absolute shrinkage and selection operator
GP: gaussian process
LR: logistic regression
PLS-DA: partial least squares discrimination analysis
ROC: receiver operating characteristics
AUC: area under the curve
DCA: decision curve analysis
