# Correction: Radiomics analysis based on CT for the prediction of pulmonary metastases in ewing sarcoma

**DOI:** 10.1186/s12880-023-01119-x

**Published:** 2023-10-13

**Authors:** Ying Liu, Ping Yin, Jingjing Cui, Chao Sun, Lei Chen, Nan Hong, Zhentao Li

**Affiliations:** 1 Department of Radiology, Peking University People’s Hospital, 11 Xizhimen Nandajie, Xicheng District, Beijing, 100044 People’s Republic of China; 2 United Imaging Intelligence (Beijing) Co., Ltd, Yongteng North Road, Haidian District, Beijing, 100094 People’s Republic of China

**Correction: Liu et al. BMC Medical Imaging (2023) 23:147**

10.1186/s12880-023-01077-4

Following the publication of the original article [1], it was brought to our attention that an error had been introduced during typesetting. In the article [1], it was stated that the Corresponding Author, Zhentao Li, was affiliated with United Imaging Intelligence (Beijing) Co., Ltd, located at Yongteng North Road, Haidian District, Beijing 100,094, People’s Republic of China.

The correct affiliation for Zhentao Li should have been the Department of Radiology, Peking University People’s Hospital, located at 11 Xizhimen Nandajie, Xicheng District, Beijing, 100,044, People’s Republic of China.

The author group information has been updated accordingly, and the original article [1] has been corrected.
